# Supplementary material for: The ataxin-1 interactome reveals direct connection with multiple disrupted nuclear transport pathways
Source: Nat Commun. 2020 Jul 3;11:3343. doi: 10.1038/s41467-020-17145-0 (PMC7334205; doi:10.1038/s41467-020-17145-0)
Supplement: Supplementary file 1 — Supplementary Information [file 41467_2020_17145_MOESM1_ESM.pdf]

Supplementary Information

The ataxin-1 interactome reveals direct connection with multiple disrupted nuclear transport pathways

Zhang et al

Supplementary Figures

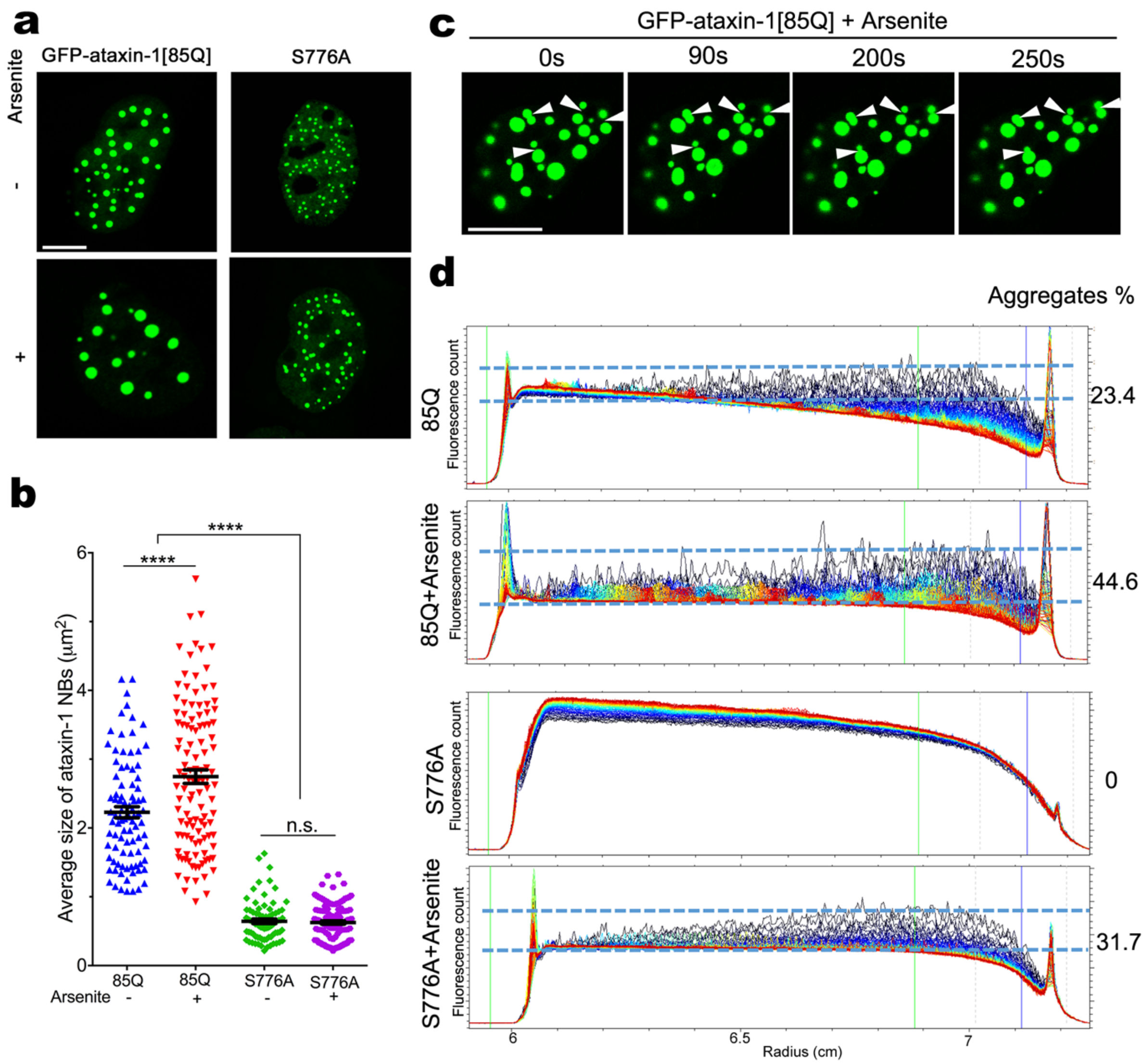

Supplementary Figure 1. Arsenite stress increases ataxin-1[85Q] nuclear body size and ataxin-1[85Q] protein aggregation in cells.

Cells were transfected to express GFP-ataxin-1[85Q] or GFP-ataxin-1[85Q]S776A (abbreviated as S776A) and treated with arsenite as indicated at 24 h post-transfection. Ad293 cells were then (a) fixed for CLSM imaging, or (c) incubated in the chamber (37 °C, 5% CO_2) for live cell imaging, or (d) lysed and sucrose added (to a final concentration of 2M) for analysis by analytical ultracentrifugation to detect GFP-ataxin-1 protein aggregation through detection of the GFP signal of the sedimenting species. (a) Representative images of cells expressing GFP-ataxin-1[85Q] or GFP-ataxin-1[85Q] S776A under control conditions or in the presence of arsenite. (b) Ataxin-1 nuclear body (NB) sizes were quantitated. Results represent the mean ± SEM (n = 100 cells analysed), p value: “85Q” vs “85Q + Arsenite” < 0.00001, “S776A” vs

“S776A + Arsenite” = 0.86437, “85Q” vs “S776A” < 0.00001, “85Q + Arsenite” vs “S776A + Arsenite” < 0.00001 (**** $p<0.0001$, n.s. not significant, one-way ANOVA and Tukey’s multiple comparisons test). Source data are provided as a Source Data file. (c) Representative live cell images of cells expressing GFP-ataxin-1[85Q] after the treatment with arsenite for > 30 min. Nuclear body fusion is denoted by arrowheads. (a&c) Scale bars = 10 μm. (d) Analytical ultracentrifugation fluorescence count analyses for GFP-ataxin-1[85Q] or GFP-ataxin-1[85Q]S776A under control conditions or in the presence of arsenite. The percentage of total fluorescence attribute to ataxin-1 aggregates was estimated from the proportion of the fast-moving sedimenting boundaries (centrifugal radius ≥ 7.1 cm) in the radial scans.

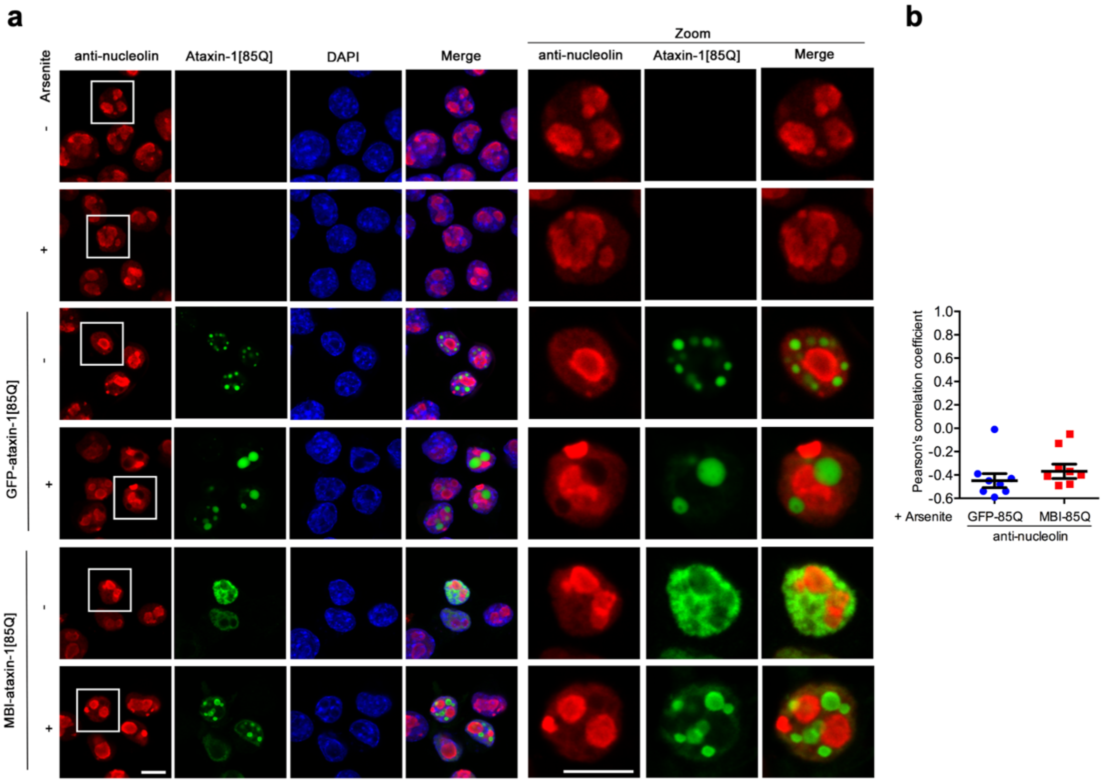

Supplementary Figure 2. Ataxin-1 nuclear bodies do not co-localise with nucleoli. Neuro-2a cells were transfected to express GFP-ataxin-1[85Q] or MBI-ataxin-1[85Q] and treated with arsenite as indicated at 24 h post-transfection. Cells were then fixed, stained with anti-nucleolin antibody together with DAPI, before CLSM imaging. (a) Representative images are shown; merge panels overlay nucleolin (nucleolus), ataxin-1[85Q] and DAPI images. Zoom images (right panels) correspond to the boxed regions. Scale bars = 10 µm. (b) The Pearson's correlation coefficient was measured for ataxin-1[85Q] and nucleolin to assess co-localisation where ≤ 0 indicates no correlation between two channels whereas 1 indicates complete correlation of two channels. Results represent the mean ± SEM (n = 9 cells imaged across 3 independent experiments) (n.s. = not significant). Source data are provided as a Source Data file.

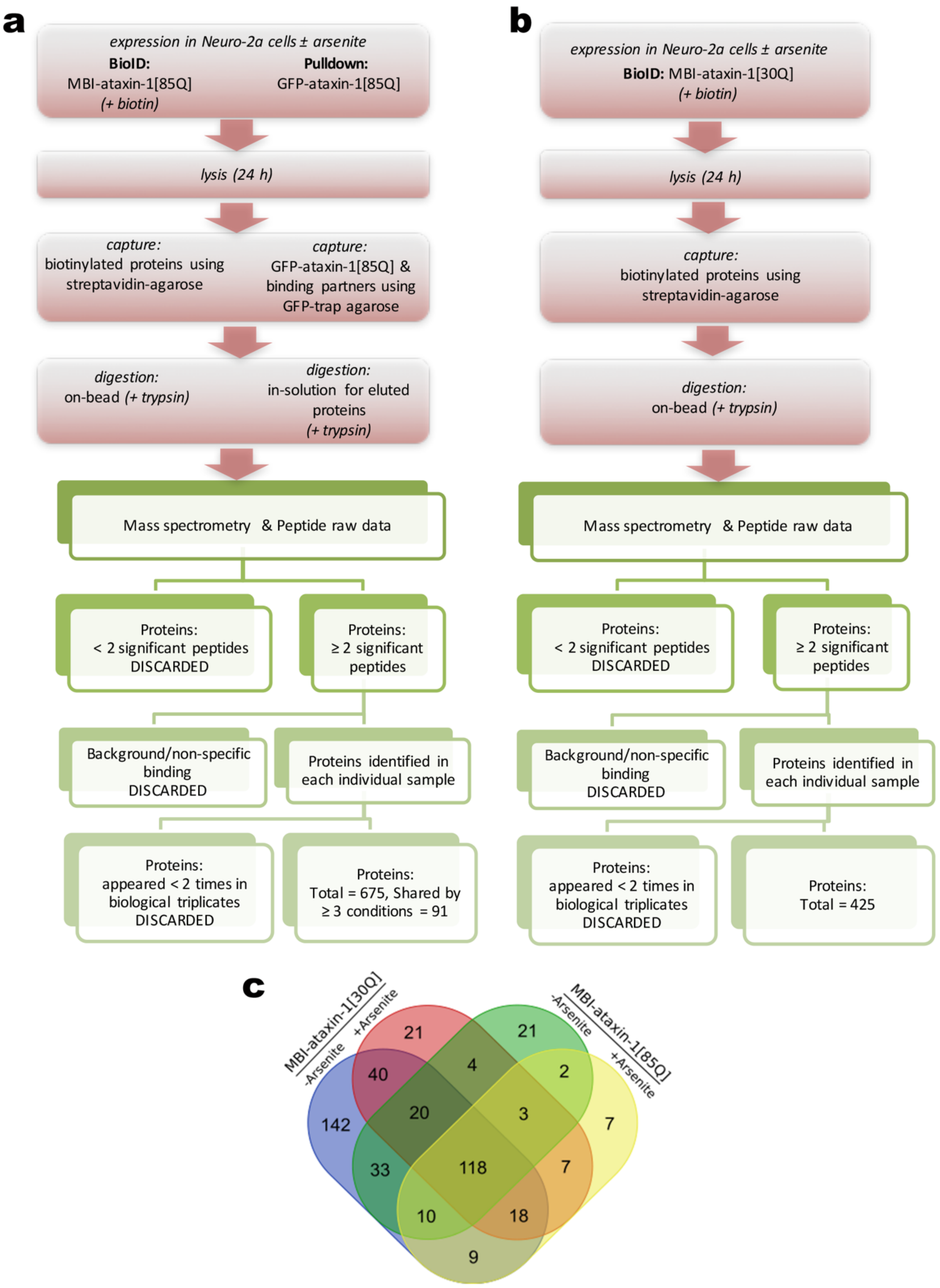

Supplementary Figure 3. Mass spectrometry workflows identify interactomes of ataxin-1[85Q] and ataxin-1[30Q].

Sample preparation for mass spectrometry to identify ataxin-1 interactomes in Neuro-2a cells (± arsenite treatment, 300 µM, 1 h) followed parallel workflows with (a) BioID and Pulldown

protocols to identify the ataxin-1[85Q] interactome or (b) the BioID protocol to identify the ataxin-1[30Q] interactome. (a-b) Cell lysis, protein capture and digestion steps were followed by mass spectrometry and peptide raw data analyses (MASCOT) that identified peptides from 4 treatment conditions (BioID ± arsenite; Pulldown ± arsenite) for ataxin-1[85Q], or 2 treatment conditions (BioID ± arsenite) for ataxin-1[30Q], that were matched to the protein reference library SWISSPROT (Mus musculus). Proteins identified by ≥ 2 significant peptides were retained, then background/non-specific binding partners removed as defined for the BioID protocol by control samples prepared from non-transfected cells incubated in the presence of biotin (50 μM) for 24 h, or for the Pulldown protocol by cells transfected to express GFP only for 24 h. The final lists, of proteins that appeared at least twice from biological triplicates, included 675 proteins for ataxin-1[85Q] or 425 proteins for ataxin-1[30Q]. (c) Venn diagram overview of the 425 identified proteins in the interactome of ataxin-1[30Q] plus the additional 30 identified proteins uniquely identified in the interactome of ataxin-1[85Q] (i.e. total protein number = 455), grouped according to cell exposure (± arsenite treatment) and expression of ataxin-1 protein (MBI-ataxin-1[30Q] and MBI-ataxin-1[85Q]).

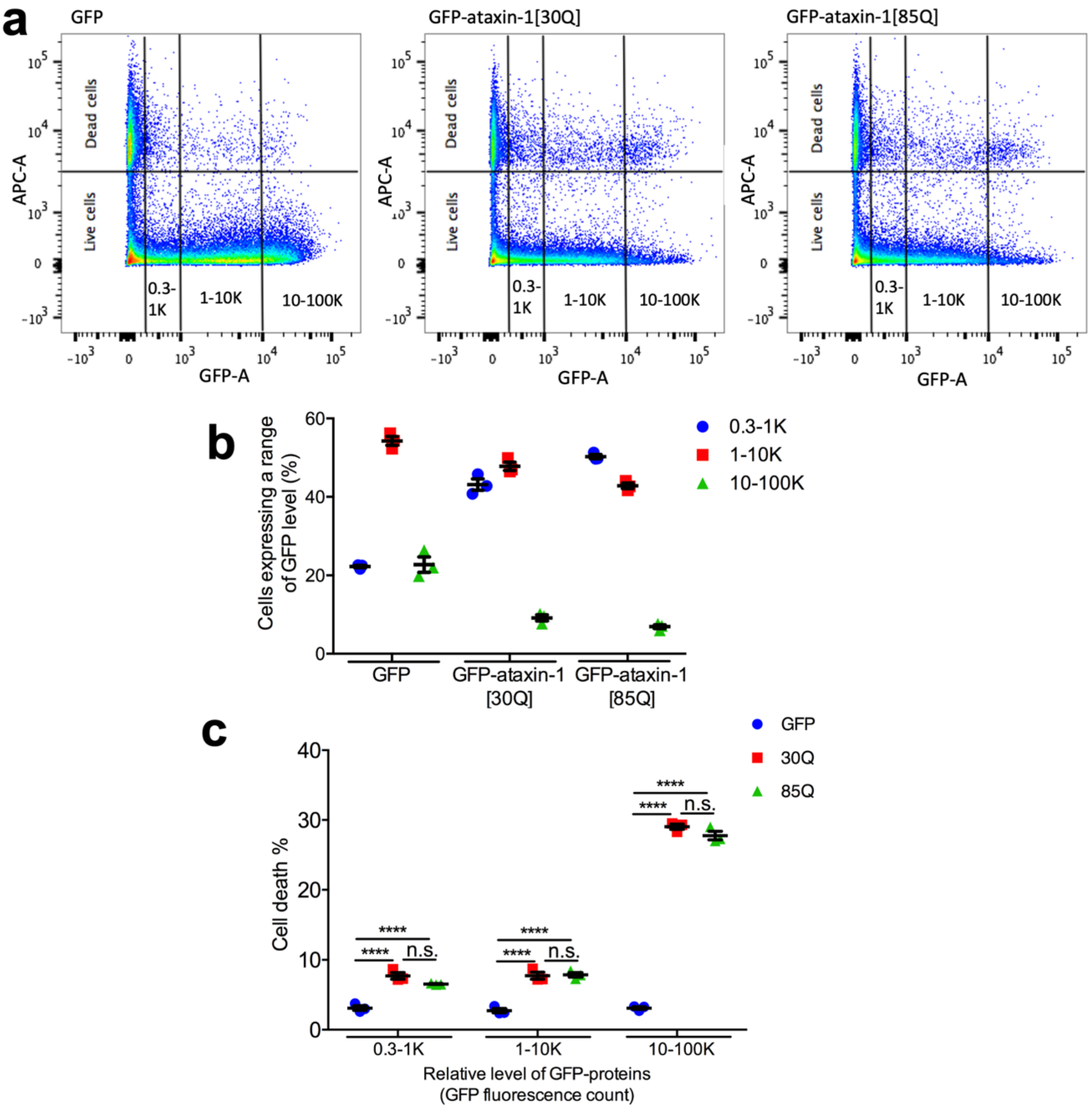

Supplementary Figure 4. Cell death is increased with higher levels of ataxin-1[30Q] or ataxin-1[85Q].

Neuro-2a cells were transfected to express GFP, GFP-ataxin-1[30Q], or GFP-ataxin-1[85Q]. At 24 h post-transfection, cells were collected into round-bottom glass tubes, and incubated with SYTOX Red dead cell stain (15 min) prior to fluorescence detection by flow cytometry (BD LSRFortessa). Cells were gated using same gating strategy provided in Supplementary Figure 5b. (a) Flow cytometry results were analyzed using FlowJo. Non-expression of GFP proteins was gated as 0-0.3K and three ranges of GFP fluorescence counts (x-axis: FITC-A) were gated and analysed: 0.3-1K, 1-10K, 10-100K. In each GFP fluorescence range, SYTOX Red was gated (y-axis: APC-A) for live cells or dead cells as indicated. (b) For each range of GFP fluorescent counts, the % cells within the population were estimated. (c) Cell death (%)

was calculated for cells with the different levels of GFP, GFP-ataxin-1[30Q], or GFP-ataxin-1[85Q] detection to allow estimates of toxicity at low (0.3-1K), moderate (1-10K) and high (10-100K) levels of GFP-tagged protein expression. Results represent mean ± SEM calculated from 3 independent experiments, p value (GFP vs 30Q, GFP vs 85Q, 30Q vs 85Q) in group 0.3-1K: $p < 0.00001$, $p = 0.00002$, $p = 0.11185$; group 1-10K: $p < 0.00001$, $p < 0.00001$, $p = 0.97280$; group 10-100K: $p < 0.00001$, $p < 0.00001$, $p = 0.08171$ (****$p<0.0001$, n.s. = not significant, two-way ANOVA and Tukey's multiple comparisons test). Source data are provided as a Source Data file.

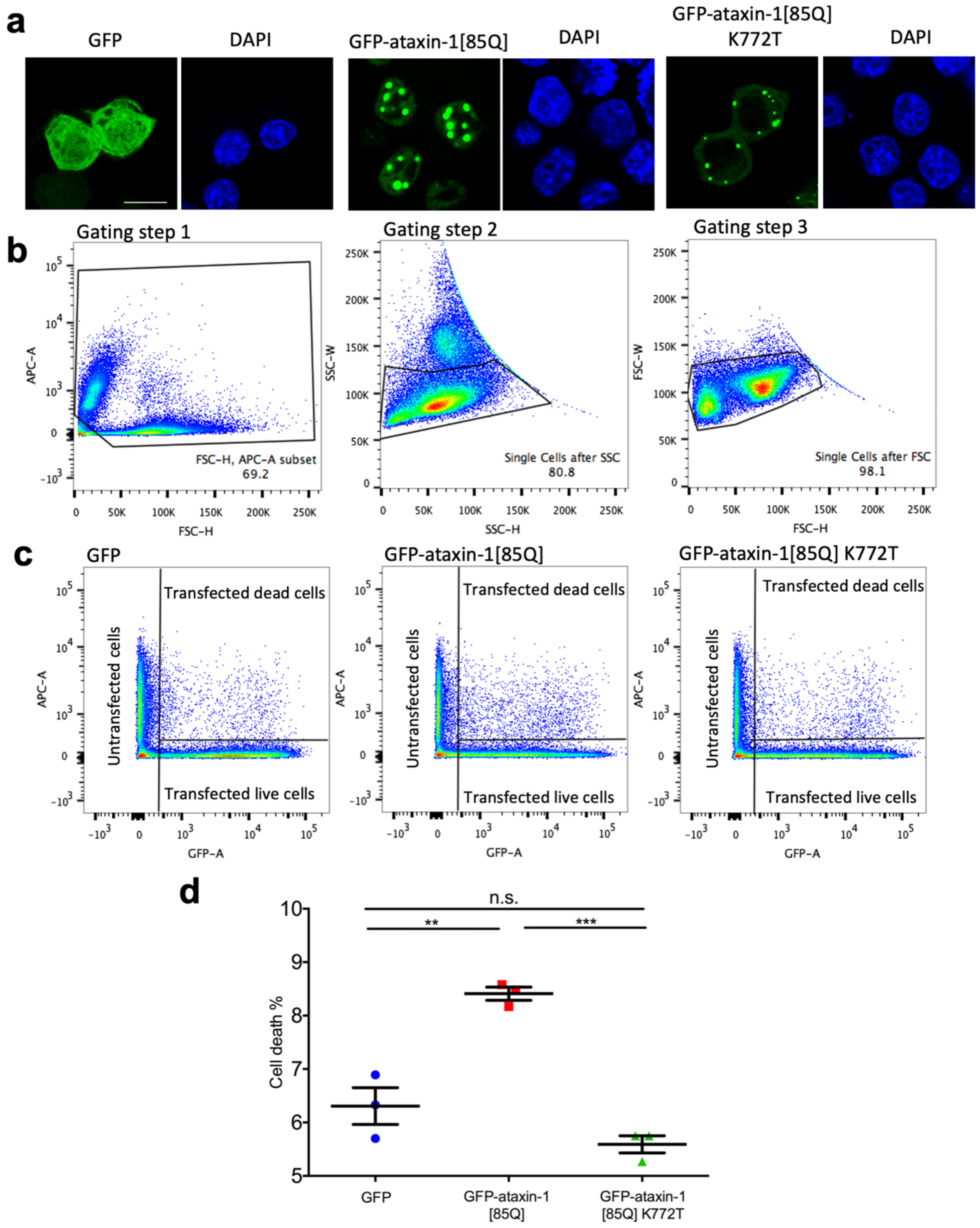

Supplementary Figure 5. Ataxin-1[85Q] mutant that does not enter nucleus shows no toxicity compared to nucleus accumulated ataxin-1[85Q]. Neuro-2a cells were transfected to express GFP or GFP-ataxin-1[85Q] or GFP-ataxin-1[85Q] (K772T). At 24 h post-transfection, cells were (a) fixed and stained with DAPI before CLSM imaging or (b) collected into round-bottom glass tubes, and added with SYTOX Red dead cell stain for 15 min prior to fluorescence detection by flow cytometry (BD LSRFortessa). (a) Representative images are

shown for different constructs localization with DAPI staining for nucleus. Scale bar = 10 μm. (b) Gating strategy used to isolate intact Neuro-2a cells. The black line contours cells that are selected from each gating step. (c)The cell populated was further gated and (d) cell death (%) was calculated for the total transfected cell population under the 3 conditions. Results represent mean ± SEM calculated from 3 independent experiments, p value: GFP vs [85Q] $p = 0.00130$, GFP vs [85Q]K772T $p = 0.69997$, [85Q] vs [85Q]K772T $p = 0.00039$ (**$p<0.01$, ***$p<0.001$, n.s. = not significant, one-way ANOVA and Tukey's multiple comparisons test). Source data are provided as a Source Data file.

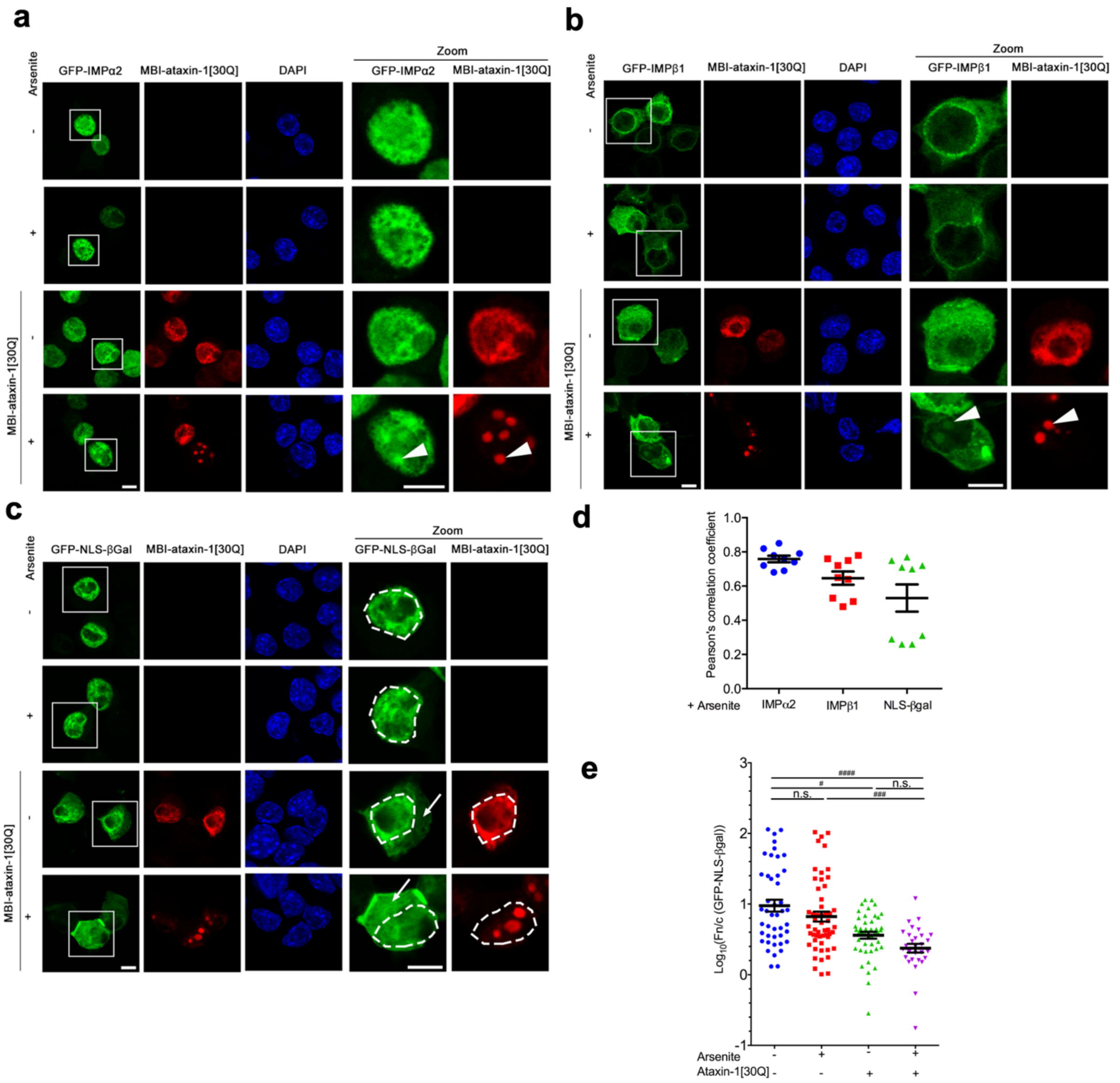

Supplementary Figure 6. Overexpression of wild-type ataxin-1[30Q] can mis-localise nuclear transporters, and disrupt the classical nuclear import pathway.

Neuro-2a cells were transfected to co-express MBI-ataxin-1[85Q] together with (a) GFP-IMPα2, (b) GFP-IMPβ1 or (c) GFP-NLS-βGal. At 24 h post-transfection., cells were treated with arsenite as indicated, and then fixed, stained with anti-myc antibody and DAPI, before CLSM imaging. Representative images of cells are shown, with zoom images (right panels) corresponding to the boxed regions. Increased mis-localisation is indicated by the white arrowheads (a-b). (c) The position of the nucleus as determined by DAPI staining is indicated by the white dashed lines; thin white arrows denote increased cytoplasmic fluorescence in the presence of MBI-ataxin-1[30Q]. Scale bar = 10 μm. (d) Pearson's correlation coefficients, calculated using the coloc2 plugin for localization between fluorophores in a 25 μm^2 square

centred over each arsenite-induced ataxin-1 nuclear body, quantitatively assessed co-localisation of GFP-tagged proteins with ataxin-1 nuclear bodies, where ≤ 0 indicates no correlation between two channels whereas 1 indicates complete correlation of two channels. Results represent the mean ± SEM (n = 9 cells imaged across 3 independent experiments). (e) Integrated fluorescence intensity in nucleus and cytoplasm was estimated and the nuclear to cytoplasmic fluorescence ratio (Fn/c) determined using a modified CellProfiler pipeline. Results represent the mean $\log_{10}$ Fn/c ± SEM calculated across 3 independent experiments using the following numbers of analysed cells: -arsenite/-ataxin-1[30Q] n = 44, +arsenite/-ataxin-1[30Q] n = 53, -arsenite/+ataxin-1[30Q] = 44, +arsenite/+ataxin-1[30Q] n = 29, p value from top to bottom and left to right: p < 0.00001, p = 0.01446, p = 0.11744, p > 0.99999, p = 0.00027 (# p< 0.05, ### p< 0.001, #### p<0.0001, n.s. not significant, Mann-Whitney and Kruskal-Wallis non-parametric test). Source data are provided as a Source Data file.

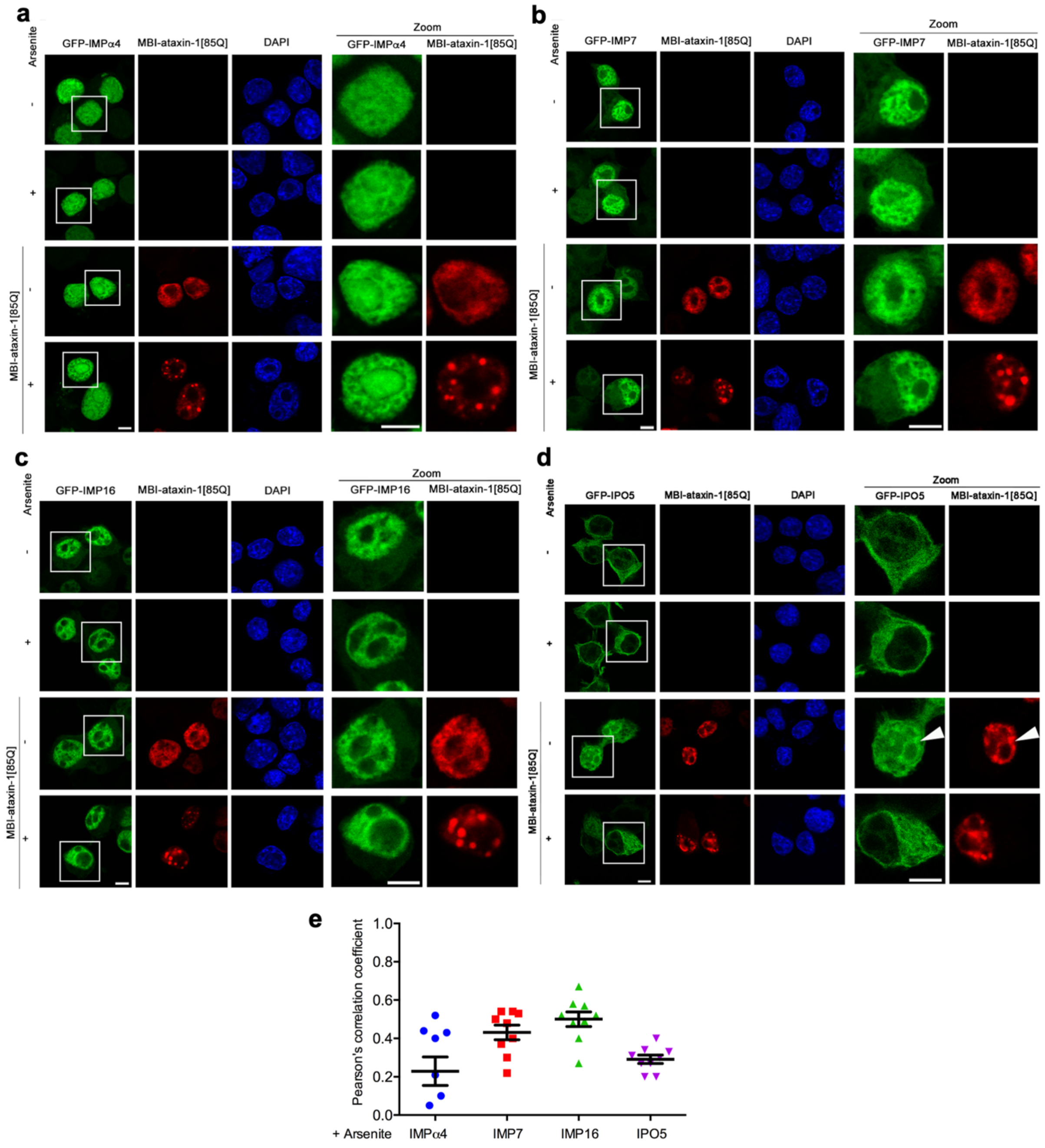

Supplementary Figure 7. Importin-α4, importin-7, importin-16 and importin-5 do not co-localise with nuclear bodies formed by polyQ-ataxin-1.

Neuro-2a cells were co-transfected to express MBI-ataxin-1[85Q] together with (a) GFP-IMPα4, (b) GFP-Importin-7 (IMP7), (c) GFP-Importin-16 (IMP16) or (d) GFP-Importin-5 (IPO5). At 24 h post-transfection, cells were treated with arsenite as indicated, and then fixed, stained with anti-myc antibody and DAPI, before CLSM imaging. Representative images are shown; zoom images (right panels) correspond to the boxed regions. (d) Mis-localisation of IPO5 is indicated by the white arrowheads. Scale bar = 10 μm. (e) Pearson's correlation

coefficients, calculated using the coloc2 plugin for localization between fluorophores in a 25 μm^2 square centred over each arsenite-induced ataxin-1 nuclear body, quantitatively assessed co-localisation of GFP-tagged proteins with ataxin-1 nuclear bodies, where ≤ 0 indicates no correlation between two channels whereas 1 indicates complete correlation of two channels. Results represent the mean ± SEM (n = 9 cells imaged across 3 independent experiments). Source data are provided as a Source Data file.

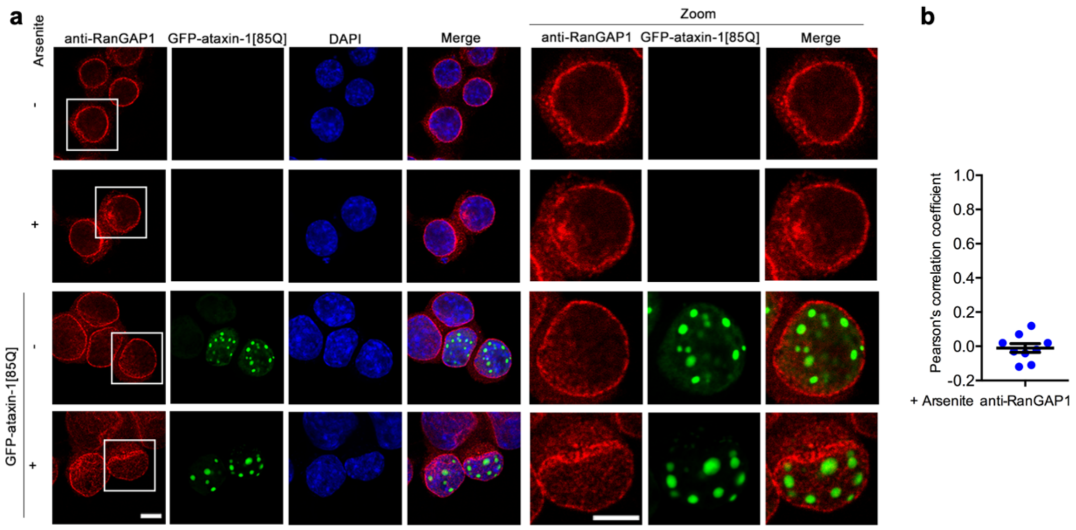

Supplementary Figure 8. RanGAP1 localisation is unaltered upon polyQ-ataxin-1 expression.

Neuro-2a cells were transfected to express GFP-ataxin-1[85Q]. At 24 h post-transfection, cells were treated with arsenite as indicated, and then fixed, stained with anti-RanGAP1 antibody and DAPI, before CLSM imaging. (a) Representative images are shown; merge panels overlay RanGAP1, GFP, and DAPI images. Zoom images (right panels) correspond to the boxed region. Scale bar = 10 µm. (b) Pearson's correlation coefficients, calculated using the coloc2 plugin for localization between fluorophores in a 25 μm^2 square centred over each arsenite-induced ataxin-1 nuclear body, quantitatively assessed co-localisation of GFP-tagged proteins with ataxin-1 nuclear bodies, where ≤ 0 indicates no correlation between two channels whereas 1 indicates complete correlation of two channels. Results represent the mean ± SEM (n = 9 cells imaged across 3 independent experiments). Source data are provided as a Source Data file.

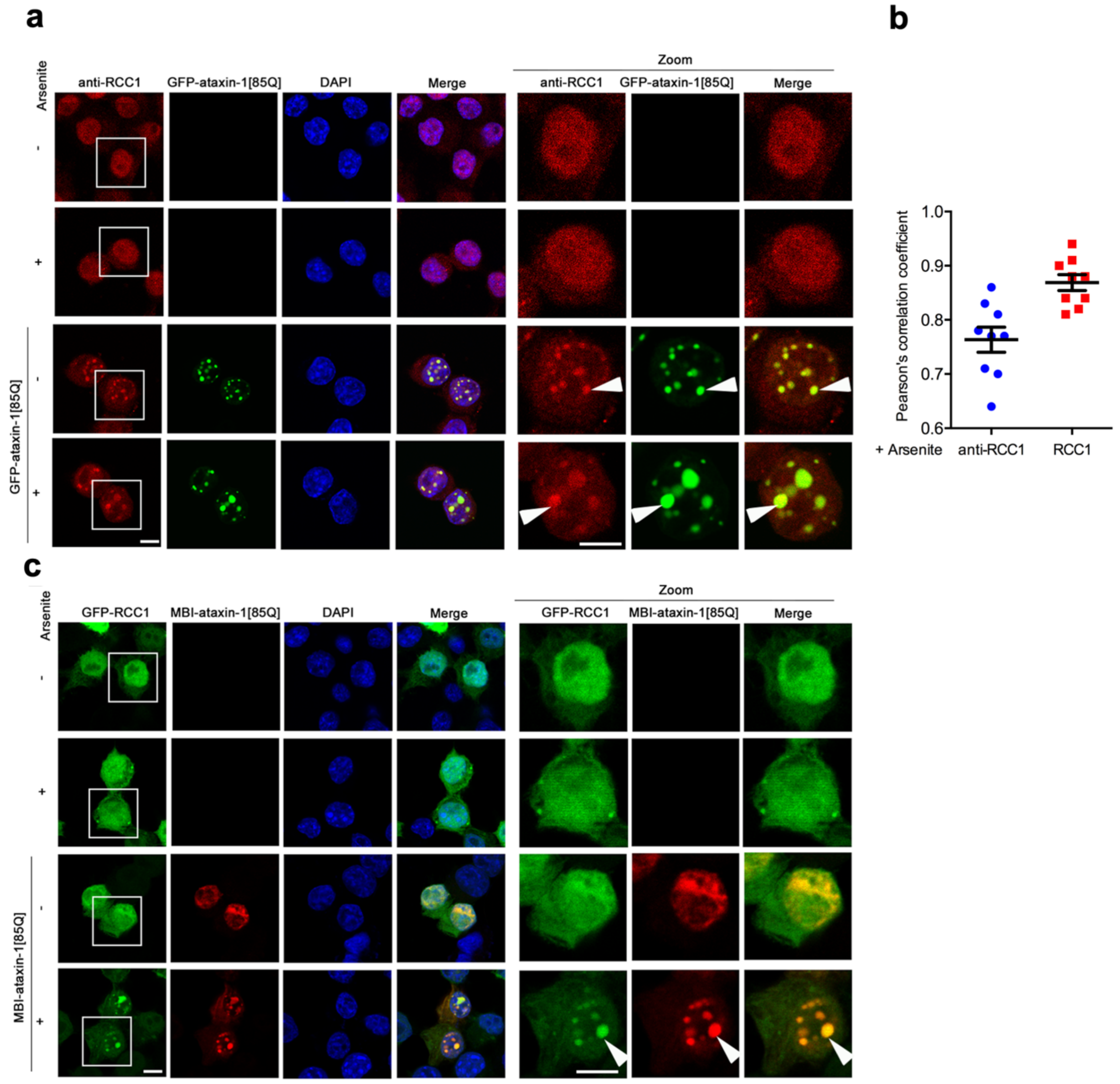

Supplementary Figure 9. RCC1 mis-localises in the presence of ataxin-1 nuclear bodies. Neuro-2a cells were (a) transfected to express GFP-ataxin-1[85Q] or (b) MB-ataxin-1[85Q] together with GFP-RCC1. At 24 h post-transfection, cells were treated with arsenite as indicated, and then fixed, stained with (a) anti-RCC1 or anti-myc (b) antibodies, and DAPI, before CLSM imaging. Representative images are shown; merge panels overlay (a) RCC1, GFP, and DAPI images (b) GFP, myc, and DAPI images. Zoom images (right panels) correspond to the boxed region; mis-localisation is denoted by the white arrowheads. Scale bar = 10 µm. (c) Pearson's correlation coefficients, calculated using the coloc2 plugin for localization between fluorophores in a 25 µm^2 square centred over each arsenite-induced ataxin-1 nuclear body, quantitatively assessed co-localisation of RCC1 proteins with ataxin-1 nuclear bodies, where ≤ 0 indicates no correlation between two channels whereas 1 indicates

complete correlation of two channels. Results represent the mean ± SEM (n = 9 cells imaged across 3 independent experiments). Source data are provided as a Source Data file.

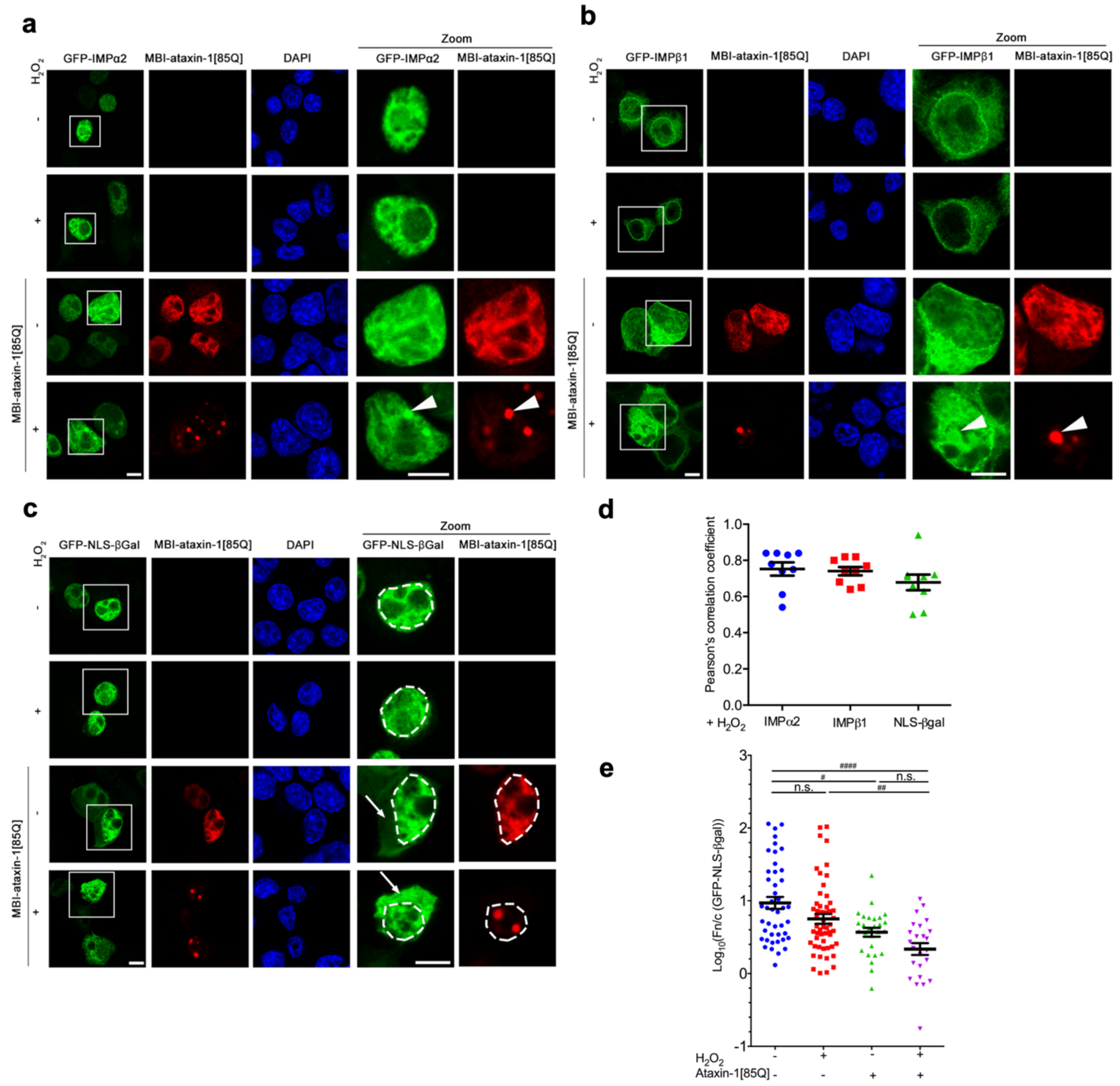

Supplementary Figure 10. H_2O_2-induced oxidative stress also influences nuclear transporter and cargo localisation in the presence of polyQ-ataxin-1.

Neuro-2a cells were transfected to co-express MBI-ataxin-1[85Q] together with (a) GFP-IMPα2, (b) GFP-IMPβ1 or (c) GFP-NLS-βGal. At 24 h post-transfection., cells were treated with hydrogen peroxide (H_2O_2) as indicated, and then fixed, stained with anti-myc antibody and DAPI, before CLSM imaging. Representative images of cells are shown, with zoom images (right panels) corresponding to the boxed regions. Increased mis-localisation is indicated by the white arrowheads (a-b). The position of the nucleus as determined by DAPI staining is indicated by the white dashed lines (c). Thin white arrows (c) denote increased cytoplasmic fluorescence in the presence of MBI-ataxin-1[85Q]. Scale bar = 10 μm. (d) Pearson's correlation coefficients, calculated using the coloc2 plugin for localization between

fluorophores in a 25 μm^2 square centred over each arsenite-induced ataxin-1 nuclear body, quantitatively assessed co-localisation of GFP-tagged proteins with ataxin-1 nuclear bodies, where ≤ 0 indicates no correlation between two channels whereas 1 indicates complete correlation of two channels. Results represent the mean ± SEM (n = 9 cells imaged across 3 independent experiments). (e) Integrated fluorescence intensity in nucleus and cytoplasm was estimated and the nuclear to cytoplasmic fluorescence ratio (Fn/c) determined using a modified CellProfiler pipeline. Results represent the mean $\log_{10}$ Fn/c ± SEM calculated across 3 independent experiments using the following numbers of analysed cells: -arsenite/-ataxin-1[85Q] n = 44, +arsenite/-ataxin-1[85Q] n = 53, -arsenite/+ataxin-1[85Q] = 27, +arsenite/+ataxin-1[85Q] n = 25, p value from top to bottom and left to right: p = 0.00003, p = 0.04867, p = 0.41441, p = 0.37664, p = 0.00875 (#p<0.05, ##p<0.01, ####p<0.0001, n.s. not significant, Mann-Whitney and Kruskal-Wallis non-parametric test). Source data are provided as a Source Data file.

Supplementary Tables

Supplementary Table 1: Nuclear transporters (IPA: RAN Signaling) identified as members of the ataxin-1[85Q] interactome.

Uniprot ID_MOUSE	Encoded protein[a]	Nuclear Transport Role
IMA1	Importin-α2	Nuclear import receptor (α-family) binding classical NLS-containing proteins; heterodimerises with importin□□1 to facilitate transit through the nuclear pore
IPO5	Importin-5/ Importin-β3	Nuclear import receptor (β-family) for proteins including some ribosomal proteins and histones
TNPO1	Transportin-1/Importin-β2	Nuclear import/export receptor (β-family) for proteins including hnRNPs, ribosomal proteins and histones
XPO1	Exportin-1/CRM1	Nuclear export receptor for diverse protein cargoes containing classical leucine-rich NES motifs
XPO2	Exportin-2/CAS	Nuclear export receptor for importin-α

[a] Includes alternative names

Supplementary Table 2: Nuclear transporters identified in both ataxin-1[30Q] and ataxin-1[85Q] interactomes

Uniprot ID_MOUSE	30Q	30Q +Arsenite	85Q	85Q +Arsenite
IMA1	Y	Y	Y	Y
IPO5	Y	N	Y	N
TNPO1	Y	Y	Y	Y
XPO1	Y	Y	Y	Y
XPO2	Y	Y	Y	Y

Supplementary Table 3: A complete list of primers

Primer name	Primer sequence
mycBioID-ataxin-1[85Q] Fw	GCGAATTCATGAAATCCAACCAAGAGCGGAGC
mycBioID-ataxin-1[30Q] Rv	GCAAGCTTCTACTTGCCTACATTAGACCGGCC
mycBioID-ataxin-1[85Q] Fw	GCGAATTCATGAAATCCAACCAAGAGCGGAGC

mycBioID-ataxin-1[30Q] Rv	GCAAGCTTCTACTTGCCTACATTAGACCGGCC
VC-ataxin-1[85Q] Fw	GGCCAAGAATTCGGAAATCCAACCAAG
VC-ataxin-1[85Q] Rv	CCGGTTGGTACCCTTGCCTACATTAGACC
Ataxin-1[85Q]mVS first strand Fw	CAGATCTCGAGCTCAAGCTTCGAATTCGAAATCCAACCAAGAGCGGAGC
Ataxin-1[85Q]mVS first strand Rv	GAAATCTTCTGTTTTTAAGTCTTCCGCCTTCTTTAGC
Ataxin-1[85Q]K772T first strand Fw	CAGATCTCGAGCTCAAGCTTCGAATTCGAAATCCAACCAAGAGCGGAGC
Ataxin-1[85Q]K772T first strand Rv	CGACCACCTCCTCGTCCTCGTTGCC
Ataxin-1[85Q]mVS second strand Fw	GTGGATCCCGGGCCCGCGGTACCCTACTTGCCTACATTAGACC
Ataxin-1[85Q]mVS second strand Rv	GACTTAAAAACAGAAGATTTCATCCAGGATGCAGAG
Ataxin-1[85Q]K772T second strand Fw	GTGGATCCCGGGCCCGCGGTACCCTACTTGCCTACATTAGACC
Ataxin-1[85Q]K772T second strand Rv	GGCAACGAGGACGAGGAGGTGGTCG
Imp-α2 ΔIBB Fw	GGCCAAGAATTCGGAACCAGGGTACTGTAAATTGG-3
Imp-α2 ΔIBB Rv	CCGGTTGGTACCGAAGTTAAAGGTCCCAGGAGC
Imp-α2/ Imp-α2 mDE amplification Fw	GGCCAAGAATTCGGTCCACGAACGAGAATG
Imp-α2/ Imp-α2 mDE amplification Rv	CCGGTTGGTACCGAAGTTAAAGGTCCCAGGAGC
Ataxin-1 transgenic mice genotyping Fw	AGGTTCACCGGACCAGGAAGG
Ataxin-1 transgenic mice genotyping Rv	AATGAACTGGAAGGTGTGCGGC
