# Supplementary material for: The ataxin-1 interactome reveals direct connection with multiple disrupted nuclear transport pathways
Source: Nat Commun. 2020 Jul 3;11:3343. doi: 10.1038/s41467-020-17145-0 (PMC7334205; doi:10.1038/s41467-020-17145-0)
Supplement: Supplementary file 3 — Description of Additional Supplementary Files [file 41467_2020_17145_MOESM3_ESM.docx]

**File Name: Supplementary Data 1**

**Description**: BioID and Pulldown protocols were used to identify ataxin-1[85Q] interactome in Neuro-2a cells. 675 identified proteins with ≥ 2 significant peptides were retained among all 4 treatment conditions (BioID ± arsenite; Pulldown ± arsenite). Column A = Uniprot ID, column B = Protein names, column C-F = 4 conditions, column H-I = summary of number of proteins in each condition. In column C-F, Y = Yes (protein is identified in the specified condition); N = No (protein is not identified in the specified condition). Different colour in rows specify the number of times the protein is identified among different conditions. Light orange = 4Y (protein identified in all 4 conditions), light green = 3Y1N (protein identified in 3 conditions), light purple = 2Y2N (protein identified in 2 conditions), grey = 1Y3N (protein identified in only 1 condition).

**File Name: Supplementary Data 2**

**Description**: BioID protocols were used to identify ataxin-1[85Q] or ataxin-1[30Q] interactome in Neuro-2a cells. 455 identified proteins with ≥ 2 significant peptides were retained among all 4 treatment conditions (85Q ± arsenite; 30Q ± arsenite). Column A = Uniprot ID, column B = Protein names, column C-F = 4 conditions, column H-I = summary of number of proteins in each condition. In column C-F, Y = Yes (protein is identified in the specified condition); N = No (protein is not identified in the specified condition). Different colour in rows specify the number of times the protein is identified among different conditions. Light orange = 4Y (protein identified in all 4 conditions), light green = 3Y1N (protein identified in 3 conditions), light purple = 2Y2N (protein identified in 2 conditions), grey = 1Y3N (protein identified in only 1 condition).
