# Supplementary material for: The ataxin-1 interactome reveals direct connection with multiple disrupted nuclear transport pathways
Source: Nat Commun. 2020 Jul 3;11:3343. doi: 10.1038/s41467-020-17145-0 (PMC7334205; doi:10.1038/s41467-020-17145-0)
Supplement: Supplementary file 6 — Reporting Summary [file 41467_2020_17145_MOESM6_ESM.pdf]

nature research

Corresponding author(s): David A. Jans

Last updated by author(s): May 30, 2020

Reporting Summary

Nature Research wishes to improve the reproducibility of the work that we publish. This form provides structure for consistency and transparency in reporting. For further information on Nature Research policies, see Authors & Referees and the Editorial Policy Checklist .

Statistics

For all statistical analyses, confirm that the following items are present in the figure legend, table legend, main text, or Methods section.

n/a	Confirmed	
☐	☒	The exact sample size (*n*) for each experimental group/condition, given as a discrete number and unit of measurement
☐	☒	A statement on whether measurements were taken from distinct samples or whether the same sample was measured repeatedly
☐	☒	The statistical test(s) used AND whether they are one- or two-sided<br>*Only common tests should be described solely by name; describe more complex techniques in the Methods section.*
☐	☒	A description of all covariates tested
☐	☒	A description of any assumptions or corrections, such as tests of normality and adjustment for multiple comparisons
☐	☒	A full description of the statistical parameters including central tendency (e.g. means) or other basic estimates (e.g. regression coefficient) AND variation (e.g. standard deviation) or associated estimates of uncertainty (e.g. confidence intervals)
☐	☒	For null hypothesis testing, the test statistic (e.g. *F*, *t*, *r*) with confidence intervals, effect sizes, degrees of freedom and *P* value noted<br>*Give P values as exact values whenever suitable.*
☒	☐	For Bayesian analysis, information on the choice of priors and Markov chain Monte Carlo settings
☒	☐	For hierarchical and complex designs, identification of the appropriate level for tests and full reporting of outcomes
☐	☒	Estimates of effect sizes (e.g. Cohen's *d*, Pearson's *r*), indicating how they were calculated

Our web collection on statistics for biologists contains articles on many of the points above.

Software and code

Policy information about availability of computer code

Data collection	Q-Exactive plus mass spectrometer (Thermo Scientific); ChemiDoc imager (Bio-Rad); BD LSRFortessa (BD Bioscience); Leica TCS SP5; XL-A analytical ultracentrifuge (Beckman Coulter, Fullerton, CA)
Data analysis	Mascot search engine (Matrix Science version 2.4); Ingenuity pathway analysis (IPA version 01-04; QIAGEN); FlowJo software (Version vX.0.7); CellProfiler cell image analysis software (version 2.1.1 for Mac); Fiji software (version 2017-05-30 for Mac); Graphpad Prism 6 (version 6.00 for Mac); ImageJ software (1.52n public domain)

For manuscripts utilizing custom algorithms or software that are central to the research but not yet described in published literature, software must be made available to editors/reviewers. We strongly encourage code deposition in a community repository (e.g. GitHub). See the Nature Research guidelines for submitting code & software for further information.

Data

Policy information about availability of data

All manuscripts must include a data availability statement. This statement should provide the following information, where applicable:

- Accession codes, unique identifiers, or web links for publicly available datasets
- A list of figures that have associated raw data
- A description of any restrictions on data availability

The authors declare that the datasets generated during and/or analysed during the current study are available via ProteomeXchange, with identifier PXD010352. All other data supporting the findings of this study are available within the Article and its Supplementary Information. The raw data generated in this study are available through the website: https://www.ebi.ac.uk/pride/archive/projects/PXD010352

Field-specific reporting

Please select the one below that is the best fit for your research. If you are not sure, read the appropriate sections before making your selection.

☒ Life sciences ☐ Behavioural & social sciences ☐ Ecological, evolutionary & environmental sciences

For a reference copy of the document with all sections, see nature.com/documents/nr-reporting-summary-flat.pdf

Life sciences study design

All studies must disclose on these points even when the disclosure is negative.

Sample size	Number of cells used for quantitative and statistical analyses were above 25 from 3 replicates to ensure the representative images shown are typical results. Consistent results were observed from randomly picked 6-7 cells in each replicate, therefore 25 cells in total were consider enough.
Data exclusions	No data were excluded from the analyses.
Replication	All experiments were prepared with biological triplicate and all attempts at replication were successful.
Randomization	For imaging experiments, each cell was randomly chosen; for other experiments randomization is not relevant as cells in bulk were used for mass spectrometry, analytical ultracentrifugation, or flow cytometry etc.
Blinding	For the quantitative study in mice, analyzers were blinded to avoid bias in phenotype scoring. For imaging experiments in cells and other in vitro experiments, there is no special blinding as the analyses were performed by machine or software where blinding is not necessary to reduce bias.

Reporting for specific materials, systems and methods

We require information from authors about some types of materials, experimental systems and methods used in many studies. Here, indicate whether each material, system or method listed is relevant to your study. If you are not sure if a list item applies to your research, read the appropriate section before selecting a response.

Materials & experimental systems

n/a	Involved in the study	
☐	☒	Antibodies
☐	☒	Eukaryotic cell lines
☒	☐	Palaeontology
☐	☒	Animals and other organisms
☒	☐	Human research participants
☒	☐	Clinical data

Methods

n/a	Involved in the study	
☒	☐	ChIP-seq
☐	☒	Flow cytometry
☒	☐	MRI-based neuroimaging

Antibodies

Antibodies used	Antibodies (1:100 dilutions) for immunofluorescence used were as follows: anti-cMyc (sc-40, Santa Cruz); anti-nucleolin (ab22758, abcam); anti-RanGAP1 (ab2081, abcam); anti-RCC1 (sc-1162, Santa Cruz); Alexa Fluor® 568-conjugated secondary antibodies (A-11004, Invitrogen, 1:300 dilution); Alexa Fluor® 488-conjugated Streptavidin (S11223, Invitrogen, 1:300 dilution); NUP62 (BD Biosciences #610498; 1:500 dilution), NUP98 (Santa Cruz #SC-74553; 1:500 dilution) or IMPβ1 (Santa Cruz #SC-137016; 1:500 dilution); anti-ataxin-1 (12nq) (Burright et al., 1995) (Ref 87 in the manuscript) (1:2000 dilution) and anti-calbindin (Santa Cruz #SC-7691; 1:250 dilution).
Validation	Antibodies were previously validated as per the manufacturer's website or published papers cited in this manuscript. Further more, in our specific experiments, anti-cMyc (sc-40, Santa Cruz), anti-nucleolin (ab22758, abcam), anti-RanGAP1 (ab2081, abcam), and anti-RCC1 (sc-1162, Santa Cruz) were validated in Neuro-2a cells (mouse cell line) by immunofluorescence. NUP62 (BD Biosciences #610498; 1:500 dilution), NUP98 (Santa Cruz #SC-74553; 1:500 dilution) or IMPβ1 (Santa Cruz #SC-137016; 1:500 dilution); anti-ataxin-1 (12nq) (Burright et al., 1995) (Ref 87 in the manuscript) (1:2000 dilution) and anti-calbindin (Santa Cruz #SC-7691; 1:250 dilution) were validated in transgenic mice by immunofluorescence.

Eukaryotic cell lines

Policy information about cell lines

Cell line source(s)	Neuro-2a cells; AD293 cells; Hela cells (from lab cultures originally obtained from ATCC)

Authentication	None of the cell lines used were authenticated
Mycoplasma contamination	The cell lines were not tested for mycoplasma contamination
Commonly misidentified lines (See ICLAC register)	Not included

Animals and other organisms

Policy information about studies involving animals; ARRIVE guidelines recommended for reporting animal research

Laboratory animals	Transgenic mice on FVB background (FVB, [85Q], [30Q]S776D, or [82Q]CIC(mVS), 12 weeks old, both male and female for each species
Wild animals	No wild animals were used in this study
Field-collected samples	No field-collected samples were used in this study
Ethics oversight	All mice were housed and managed by Research Animal Resources (RAR) under specific pathogen–free conditions in an Association for Assessment and Accreditation of Laboratory Animal Care International (AAALAC)–approved facility. Ethic oversight and animal use protocol approvals were provided by the University of Minnesota Institutional Animal Care and Use Committee (IACUC).

Note that full information on the approval of the study protocol must also be provided in the manuscript.

Flow Cytometry

Plots

Confirm that:

- [x] The axis labels state the marker and fluorochrome used (e.g. CD4-FITC).
- [x] The axis scales are clearly visible. Include numbers along axes only for bottom left plot of group (a 'group' is an analysis of identical markers).
- [x] All plots are contour plots with outliers or pseudocolor plots.
- [x] A numerical value for number of cells or percentage (with statistics) is provided.

Methodology

Sample preparation	Neuro-2a cells (2 x 10^5 cells/well) transfected to express GFP-ataxin-1[30Q], GFP-ataxin-1[85Q], or GFP alone as a control, were suspended in PBS and transferred to 5ml polystyrene round bottom tubes (BD Bioscience). To determine the numbers of dead cells per sample, the SYTOX Red dead cell stain (ThermoFisher Scientific) was added to cell suspensions, and incubated at room temperature for 15 min before flow cytometry analysis according to the manufacturer's instructions.
Instrument	BD LSRFortessa (BDBioscience)
Software	FlowJo software (Version vX.0.7)
Cell population abundance	50,000-75,000 cells were recorded by the cytometer for each sample; all samples were prepared and measured in triplicate.
Gating strategy	The gating of debris and intact Neuro-2a cells was based on forward scatter-area (FSC-A) and the side scatter-area (SSC-A). The gating for transfected cells was based on fluorescence FITC-A (GFP, Green channel); the gate for dead cells was based on fluorescence APC-A (SYTOX, Red channel). Further gating was applied to the transfected cells based on different fluorescence counts from the green channel: 0.3-1K, 1-10K, 10-100K.

- [x] Tick this box to confirm that a figure exemplifying the gating strategy is provided in the Supplementary Information.
